# Inside the STEM pipeline: Changes in students’ biomedical career plans across the college years

**DOI:** 10.1126/sciadv.abe0985

**Published:** 2021-04-30

**Authors:** Emily Q. Rosenzweig, Cameron A. Hecht, Stacy J. Priniski, Elizabeth A. Canning, Michael W. Asher, Yoi Tibbetts, Janet S. Hyde, Judith M. Harackiewicz

**Affiliations:** 1 University of Georgia, Athens, Georgia 30602, USA.; 2 University of Texas at Austin, Austin, Texas 78712, USA.; 3 Michigan State University, East Lansing, Michigan 48824, USA.; 4 Washington State University, Pullman, Washington 99614, USA.; 5 University of Wisconsin-Madison, Madison, Wisconsin 53706, USA.; 6 University of Virginia, Charlottesville, Virginia 22904, USA.

## Abstract

Researchers often invoke the metaphor of a pipeline when studying participation in careers in science, technology, engineering, and mathematics (STEM), focusing on the important issue of students who “leak” from the pipeline, but largely ignoring students who persist in STEM. Using interview, survey, and institutional data over 6 years, we examined the experiences of 921 students who persisted in biomedical fields through college graduation and planned to pursue biomedical careers. Despite remaining in the biomedical pipeline, almost half of these students changed their career plans, which was almost twice the number of students who abandoned biomedical career paths altogether. Women changed plans more often and were more likely than men to change to a career requiring fewer years of post-graduate education. Results highlight the importance of studying within-pipeline patterns rather than focusing only on why students leave STEM fields.

## Teaser

STEM retention efforts need to go beyond “pipeline leaks” to focus on those who remain in STEM but change career plans.

## INTRODUCTION

Researchers and policy makers often invoke the metaphor of a pipeline when studying participation in careers in science, technology, engineering, and mathematics (STEM). Ideally, students who begin their college education with an interest in STEM fields will flow through this pipeline and eventually pursue STEM careers, but some students “leak” out by choosing non-STEM majors, pursuing non-STEM career paths, or dropping out of college altogether (*1*–*3*). This metaphor is widely used in contemporary research and practice, with the goal of exploring and preventing leaks in the pipeline (*2*, *4*–*7*). However, this singular focus on leaks ignores the experiences of students who remain in the STEM pipeline. To date, most research has not focused on the career paths of students who continue moving through the pipeline, because these students are assumed to be “on track.” Such an assumption warrants scrutiny. STEM fields encompass dozens of career paths, each of which has different levels of educational requirements, representation of women and people of color, salary implications, and societal demands. Not all students who initially choose a particular STEM career path ultimately pursue it. In fact, more students might change career paths within STEM fields than leave STEM fields altogether. To promote retention in particular STEM careers, then, it is essential to consider not only those who leave STEM but also those who remain in STEM fields but change career plans. For example, the Association of American Medical Colleges points to a growing shortage of physicians, a situation made worse by the coronavirus disease 2019 (COVID-19) crisis (*8*). Every student who drops their medical school aspirations in favor of a biomedical career requiring less education (e.g., to become a bachelors-level biology laboratory technician) exacerbates this critical shortage.

Why might students change career plans within the STEM pipeline? Eccles (*9*) has advanced an expectancy-value theory to account for students’ academic motivation and choices. In this model, two primary factors determine students’ motivation for making academic choices: the extent to which a student values a particular career (i.e., they perceive it to be interesting, useful, or personally meaningful) and the extent to which that student expects to succeed in that career path (*10*). College is a critical transition point for the development of students’ career values and expectancies; during this time, students explore different career options and consider how they fit with their values and competencies. The corresponding changes in values and expectancies for different careers can be positive or negative forces. Some students may change career plans because they discover that they are interested in a different career than originally intended or they believe that they will be more successful in a different career path; that is, they develop positive task values or expectancies that attract them to a new career. However, other students might realize that the day-to-day life of their intended career is boring or believe that they cannot get into graduate school for their chosen career; that is, they develop negative values or expectancies for their original career plan and become disenchanted with it. All students who change career plans likely think about both attraction and disenchantment to some extent when changing plans, but previous research suggests that many students tend to describe one type of motivation as stronger than the other when they leave STEM fields of study (*11*, *12*). In terms of attrition out of STEM fields, previous research shows that a majority of students report leaving primarily because of disenchantment with STEM career plans (*11*–*13*).

In the present study, we explored patterns of change within the STEM pipeline and examined whether students who change career plans within STEM fields do so for the same reasons as students who leave STEM career paths altogether. It is not clear whether students who remain in STEM fields but change career plans also perceive disenchantment as the primary reason for change. If students remain in STEM fields, perhaps they experience less disenchantment with their original career plan, and instead focus on the attractive aspects of alternative STEM careers. If true, policy makers hoping to encourage people toward particular STEM careers with shortages may want to rethink retention efforts. If students perceive a change in career goals as being due to attraction to a new career path within STEM, then trying to prevent disenchantment in the original career goal may not be the most effective way to retain them. Instead, it might be useful to emphasize the attractive aspects of various STEM careers (with particular attention to careers where there are shortages) and help students explore different options within STEM to find the best fit with their values and competencies.

In addition, we explored whether there were demographic differences in patterns of change in career plans within the STEM pipeline. Research indicates that students from underrepresented ethnic minority (URM) groups and women are more likely to leave some STEM fields, and a considerable body of research has examined these problems (*13*–*17**)*. However, there may be problems within the pipeline as well. It is important to determine whether there are systematic differences in who changes plans within STEM fields as a function of gender or ethnicity. Given the differences in educational requirements, salary, job availability, and societal needs for different STEM careers, the question of different trajectories inside the pipeline is critically important to promote equitable participation as students make STEM career decisions.

As an illustrative case of change within the pipeline, we focus on within-STEM career changes in one STEM subfield: the biological and medical sciences (i.e., biomedical fields). This longitudinal study examined trajectories for college students who remained in the biomedical pipeline through graduation. We started with a sample of 1193 students who had enrolled in a large introductory biology course (typically taken in the first or second year of college) at a U.S. Midwestern university between fall 2011 and spring 2014, and who intended to pursue a biomedical field of study. This course is a pre-requisite for 34 biomedical majors and a critical gateway course for pre-medical preparation. Among students who remained in the pipeline through to their career plans, 75% had begun the course with doctoral-level career aspirations (e.g., pre-med and pre-vet), with the majority of these students planning to attend medical school. We collected surveys and institutional records throughout college and then interviewed each student about their future career plans near graduation to determine if their career plans had changed and, if so, how and why their plans had changed.

## RESULTS

**Fig. 1:**
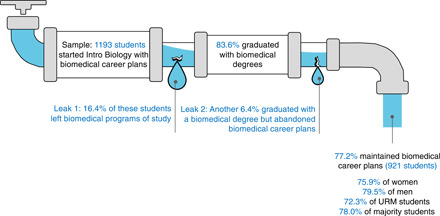
Biomedical pipeline in the current study: Attrition occurs at two points. Model based on sample of 1193 introductory biology students interviewed at time of graduation. Sample comprised 763 women and 430 men. One hundred seventy-three students were from URM groups, and 1020 students were from racial/ethnic majority groups.

We first examined whether students remained in the biomedical pipeline throughout college, measured in terms of (i) whether they graduated with a degree in a biomedical field and (ii) whether they continued to pursue a biomedical career after graduation (Fig. 1). Of 1193 participants, 997 (83.6%) graduated with a biomedical degree, 4 students (0.3%) were originally on biomedical career tracks but had not graduated as of May 2020, and 192 students (16.1%) had left biomedical fields by the time of expected graduation. Of the 997 students who graduated with a biomedical degree, 76 students (6.4% of initial sample) had abandoned biomedical career plans by graduation. The remaining 921 students (77.2% of initial sample) continued to aspire to biomedical careers after graduation.

There were no significant differences in rates of biomedical graduation or continued biomedical career plans as a function of gender. However, consistent with previous research, individuals from URM groups (African American, Hispanic/Latinx, Native American) were significantly less likely to graduate with a biomedical degree (76.9%) compared to individuals from White or Asian/Asian American (i.e., racial/ethnic majority) groups (84.7%), χ^2^(1) = 6.60, *P* = 0.010. The difference for continued biomedical career plans (72.3% URM students, 78.0% majority students) was not significant, χ^2^(1) = 2.81, *P* = 0.094.

The group of 921 students (62.9% women, 13.6% URM) who remained in the biomedical pipeline through graduation and continued to pursue biomedical career plans constituted the primary sample for our within-pipeline analyses. We first examined whether these participants had changed their career plans, from the biology class through the interview near graduation. If plans did change, we coded what type of change students made, in terms of the amount of post-graduate education required for their new career plans, compared to their career goals reported at baseline. Students were classified into one of four categories: (i) did not change career plans or (ii) changed plans to a career that required the same amount of education, (iii) fewer years of education, or (iv) more years of education. Last, we coded students’ motivations for changing plans, in terms of whether students changed plans because of disenchantment (i.e., referencing negative features of original career plan), attraction (i.e., positive aspects of their new plans), or both (*13*). This coding system was based on expectancy-value theory and characterized students’ reflections about changed plans in terms of their expectancies for success and task values for the original and new career plans. See Table 1 for examples.

**Table 1:** Aspects of career plan changes coded from interviews. *N* = 422 students who changed their career plans within biomedical fields. Forty-six students gave responses too vague to classify in terms of attraction versus disenchantment.

**Types of career plan change within biomedical fields**		
	**Frequency**	**Sample responses**
Changed to career that involves same amount of education	228	“I was pre-med when I took Biology my freshman year. I alternated between pre-med/dental and getting my Ph.D. until I looked at the salaries and quality of life of the 3 professions and chose dentistry.” “I was originally pre-med, until I realized that I am not a patient contact person and would rather teach and do research. An independent project for class made me realize that I would rather earn a Ph.D. than an M.D..”
Changed to career that requires more education	37	“Changed from maybe physician assistant school to med school.” “I discovered the M.D./Ph.D. program through my advisor and The P.I. of the lab that I worked in as a research assistant. I decided to commit to it my senior year.”
Changed to career that requires less education	157	“I realized I didn’t want to go to medical school because of the time and cost. I also realized I couldn’t have the life balance I wanted if I was a physician. I chose to go the Nurse Practitioner route for these reasons.” “I still wanted to work in the private sector but I wanted to get a Ph.D. which in the past year and a half I realized I didn’t really want.”
**Was change in career plans due to attraction or disenchantment?**		
	**Frequency**	**Sample responses**
Disenchantment with original career path	131	“I intended on going to dental school. After applying and not getting any acceptances, I began to reevaluate my future plans. This made my senior year full of stress and anxiety. I eventually decided to apply to chiropractic school.” “I wanted to go to medical school – my plans changed when my sister got sick and spent a year and a half in the hospital. Spending that much time in that environment made me realize the constraints of working in the field.”
Attraction to new career path	186	“I previously wanted to go to medical school, but decided that a I would prefer a pharmacist lifestyle, and I was more interested in the drug aspect of the medical field.” “I originally thought I would do nursing but then realized I wanted a career that required the use of more science.”
Both disenchantment and attraction	59	“I went from wanting to be an OB-GYN to wanting to be a midwife. I decided that I didn’t want to go to medical school and that midwifery is better suited toward my personality.” “I planned on going to medical school and becoming a doctor. Now I am Pre-P.A. and plan on being a physician assistant. I changed my mind because I do not want to be in school for as long as medical school takes and I want a career that offers more time to have my own life.”

**Fig. 2:**
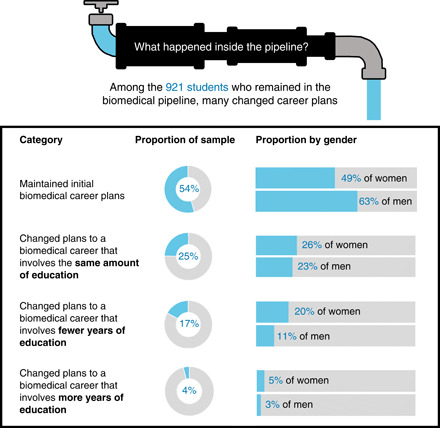
Changes in career plans among the 921 students who remained in the pipeline and maintained biomedical career plans. Sample comprised 579 women and 342 men. One hundred twenty-five students were from URM groups, and 1020 students were from majority groups.

The results reveal substantial variability in the paths students took to choosing their post-graduation biomedical careers. Almost half (46%) of the 921 students remaining in the pipeline reported having changed career plans during college, whereas 54% maintained the same plans reported at baseline. Women were more likely to have changed their career plans (51%) than men (37%), χ^2^(1) = 16.53, *P* < 0.001. URM students were not significantly more likely to change plans (53%) than majority students (45%). Figure 2 shows the patterns of change. Of the students continuing in the pipeline, 25% changed to career goals requiring the same amount of post-graduate education, 17% changed to career goals requiring fewer years of education, and 4% changed to career goals requiring more years of education. Thus, among the 422 students who changed plans, 54% made a lateral change to a career requiring the same amount of education and another 37% changed to a career requiring fewer years of education.

We tested whether URM students or women were more likely to make certain types of career plan changes and found that women were significantly more likely than men to change to career plans that involved fewer years of education, χ^2^(1) = 12.30, *P* < 0.001. Most of this change was away from doctoral-level pursuits. Among students who had reported doctoral-level career plans at baseline, 74.0% of women continued to pursue doctoral plans at graduation compared to 86.3% of men, and this gender difference was significant, χ^2^(1) = 14.87, *P* < 0.001. In contrast, there was no difference for URM (78.4%) compared to majority (78.9%) students.

What do shifts to careers that require fewer years of education look like? We conducted a case-study analysis of those who lowered their educational aspirations away from the most common career goal in this sample: medical school (46.7% of the 921 students in the sample started with this goal). Ninety-nine students (23.0%) who began with aspirations to attend medical school changed plans to careers requiring fewer years of education. The most frequent new career paths were to become a physician assistant (30 students), nurse, nurse anesthetist, or nurse practitioner (15 students), biomedical researcher at a level lower than Ph.D. (10 students), or to do a management job in the natural sciences (7 students). As in the overall analyses, women who started with medical school plans were more likely to change plans to a career requiring less education compared to men (28.2% of women; 15.4% of men), χ^2^(1) = 9.65, *P* = 0.002. Women often switched to the careers of physician assistant (36.1% of women who lowered their plans away from medical school; 14.8% of men) or nurse (18.2% of women; 7.4% of men), whereas men often switched to natural sciences manager (22.2% men; 1.4% women) or biomedical researcher (18.5% men; 6.9% women).

Next, among all students who changed plans within the pipeline (*N* = 422), we examined their motivations for changing. The interviews revealed that 50.5% of students whose responses could be classified reported that their change was at least partially due to disenchantment with their original career goal, whereas 49.5% only mentioned factors that attracted them to their new career plan. These interpretations differed significantly as a function of type of change, χ^2^(2) = 30.29, *P* < 0.001: Participants who changed plans to a career requiring fewer years of education were significantly more likely to report changing plans due to disenchantment (68.8%) versus attraction (31.2%), χ^2^(1) = 39.73, *P* < 0.001. The opposite was true for those who changed plans to a career requiring the same amount of education (attraction, 59.9%; disenchantment, 40.1%), χ^2^(1) = 16.19, *P* < 0.001, or who changed to a career requiring more education, χ^2^ (1) = 4.50, *P* = 0.034. There were no significant differences in motivations as a function of gender or URM status. For these analyses, students who reported both attraction and disenchantment are classified with the “disenchantment” group, but omitting them from this group yielded the same results.

Last, we compared the students who changed plans within the pipeline to students who left biomedical career paths altogether. There were many more students who changed career plans within biomedical fields (*N* = 422) than who had abandoned biomedical career paths by expected graduation (*N* = 272). In addition, we observed ethnic differences, but no gender differences, in attrition out of biomedical fields. This is the opposite of what we observed for within-biomedical career plan changes: gender differences, but no ethnic differences. Last, a previous study (*13*) examined retention in the pipeline at an earlier time point for this group, when 1001 students still remained in biomedical fields of study, and 192 students had left, and explored those students’ reasons for leaving biomedical fields. Comparing data from this sample to that one, 74.8% of students who left the pipeline altogether stated that their change was at least partially due to disenchantment with their original career plan. This number is significantly higher than what students who switched plans within the biomedical pipeline reported (50.5%), χ^2^(1) = 25.51, *P* < 0.001.

## DISCUSSION

Our analysis of the trajectories of students who remained in the biomedical pipeline through college graduation provides new insights about motivational dynamics inside the pipeline and demonstrates the importance of understanding this group if we are to promote retention in particular STEM careers. The sheer number of changes made by students who remained in the biomedical pipeline highlights the divergence of paths students take in their career decision-making. Almost half of the students who remained in the pipeline chose to pursue different careers than originally intended. The number of students who made within-pipeline changes was almost twice the number of students who left the pipeline altogether. Previous research has focused on the “leavers,” but our research suggests that we should also study the “stayers” more carefully, because so many of them change plans. We should not simply assume (as the pipeline metaphor seems to suggest) that students are staying on course and progressing smoothly toward intended careers just because they have not left the pipeline. To address shortages in certain STEM careers, such as the shortage of physicians in the United States, it may be fruitful to focus retention efforts on students who are still in the pipeline but considering other biomedical careers, rather than focusing exclusively on preventing students from leaving biomedical fields.

How might we accomplish this goal? Our findings indicate that students who changed career plans within biomedical fields were significantly less likely to report being motivated by disenchantment with their original plan than those who left biomedical career paths altogether; instead, they were more often motivated by attraction to alternatives within the biomedical fields. Accordingly, efforts to broaden participation in STEM should encourage career exploration by exposing students to the wide variety of careers in STEM and what makes different STEM careers attractive (e.g., importance to society, interesting and rewarding work, and work-life balance). Educators and administrators might attempt to increase interest in STEM careers with shortages by emphasizing the attractive qualities of these careers that may be underappreciated by undergraduates. Of course, we should not abandon efforts to reduce students’ feelings of disenchantment—after all, half of students who changed career paths within biomedical fields did report some disenchantment. Efforts to promote the attractiveness of certain careers should occur in addition to efforts to reduce disenchantment, not in place of them.

These efforts are all the more important given that our findings reveal new patterns of demographic differences in biomedical career pursuit. We conducted two kinds of analysis in this study: attrition analysis, using the pipeline model, and within-pipeline trajectory analysis. With respect to attrition from biomedical fields, our results are consistent with previous research (*13*–*16*): URM students were more likely to leave biomedical fields, but there were no gender differences in leaving. These findings might suggest that there is no gender imbalance in the biomedical pipeline. Consistent with national trends (*14*), women were in the majority in our sample (as in the original introductory biology class), and a greater number of women than men aspired to doctoral-level degrees in this study. However, our within-pipeline results highlight a gender difference in STEM career pursuit that has gone undetected in previous research. That is, a larger percentage of women (51%) than men (37%) changed career plans within the biomedical pipeline and, in particular, changed to careers requiring fewer years of education (resulting in a smaller percentage of women maintaining doctoral-level career aspirations).

These results illustrate that despite parity in biomedical graduation rates, women may be less likely to persist in doctoral-level biomedical career plans than men. Such changes likely occur because of the value women place on different STEM careers. Research shows that women are socialized to perceive certain careers as having less value or being a poorer fit for them, particularly when careers do not seem to afford communal values or work-life balance (*18*–*20*). As women progress through college and learn more about their career options, such socialization experiences may affect their decisions about what career paths seem to fit best with their values. Our case-study analysis of pre-medical students supports this hypothesis, as women more often shifted to careers such as physician assistant and nurse, which are still related to medicine but are generally perceived to offer better work-life balance. To recruit all available talent to high-demand STEM careers, and to ensure that gender biases are not affecting students’ career decisions, researchers and policy makers must consider how value-related factors such as work-life balance might cause women to become disenchanted with certain career paths and/or attracted to alternative paths within STEM.

Understanding who leaves STEM fields during college is critical for promoting diverse participation in STEM careers. However, it is also critically important to consider the variety of patterns among students who do not leave STEM fields. A within-pipeline analysis offers new insights into students’ academic trajectories as they prepare for careers in biomedical fields. By shining a light into the STEM pipeline, we discovered patterns of change that may help us to address critical career shortages and help more students fulfill their career goals.

## MATERIALS AND METHODS

### Participants

Participants were 1193 students who were enrolled in a large introductory biology course at a U.S. Midwestern university between fall 2011 and spring 2014 and who consented to complete an interview about their future career plans between spring 2014 and summer 2017. This group of students was 64.0% female, 72.5% White, 13.0% Asian/Asian American, and 14.5% members of underrepresented ethnic minority groups (i.e., URM, African American, Hispanic/Latino/a, or Native American). We initially obtained data from 1265 students, but only 1193 indicated baseline interest in biomedical fields while enrolled in the biology course and thus constituted the initial sample for this study (see the “Measures” section for more information). The project was approved by the University of Wisconsin-Madison Educational/Social and Behavioral Sciences Institutional Review Board, and informed consent was obtained from all participants.

These students were 64.9% of a broader sample (1837) of students who had participated in one of two research projects exploring motivation and performance in introductory biology courses; students took the course between fall 2011 and spring 2014 (*21*–*22*). In collecting follow-up interviews, we aimed to recruit as many students as possible who had participated in the two original research projects, and the research team attempted to contact all 1837 students; the vast majority of nonparticipants did not respond to the request for an interview (as opposed to actively declining participation). We obtained a sample of students that was representative of both projects’ original samples in terms of demographic variables and course achievement (see Supplementary Text for more information). The original projects tested different types of interventions in introductory biology courses that aimed to enhance students’ motivation for learning biology; the effects of such interventions have been reported in previous papers (*17*, *21*–*22*). In the present study, we combined samples of students and thus included in our sample a subset of participants from the intervention and control conditions in both research projects. The goal in combining samples was to maximize the sample size for our analysis of students’ interviews and STEM trajectories. Other research, which examined attrition out of STEM fields using the same sample of students, has used a similar approach (*13*).

As a robustness check, we ran all the analyses reported in this article controlling for which research project students took part in as well as which experimental condition students completed during the previous research projects; results returned the same pattern of results as is reported in the main text; see Supplementary Text for complete output and description.

### Procedure

As part of the two broader research projects, students completed questionnaires at the beginning and end of the introductory biology course assessing their intended majors and professional career goals. We obtained institutional records—students’ academic major history, graduation information, and course-taking details—for the semester during which they took the biology course and for any subsequent semesters up through their graduation or May 2020.

Between spring 2014 and spring 2017, participants were contacted to complete a follow-up interview about their future career plans and academic majors. We aimed to contact all participating students as close to their graduation as possible, based on when records indicated they were most likely to graduate. Because students completed their studies at different rates and took the course in different points in their schooling, the time that elapsed between taking the introductory biology course and the interview differed between students (*M* = 5.73 semesters, *SD* = 1.16, range = 2 to 9 semesters). During the interview, students responded to a set of open-ended questions about whether or not they had graduated, what their future plans were, both immediately after graduation and 10 years into the future, whether these future plans had changed since students were enrolled in the introductory biology course, and why plans changed if they did change (see supplementary tables). They responded either by phone or by typing responses to open-ended questions in an online survey system. All interviews conducted by phone were transcribed, and coding was done using the transcriptions.

### Measures

All variables discussed below were measured with students’ gender and ethnicity masked to researchers.

#### Determining baseline interest in biomedical fields

Only students with a baseline interest in biomedical fields were part of our initial sample. To determine baseline interest in biomedical fields, we used two metrics. First, on the baseline questionnaires, students were asked to write in their intended majors, which we classified as biomedical or not using a scheme developed in previous research (*17*). Second, on the baseline questionnaires, students were asked to check one or more boxes to indicate if they planned to pursue any of five doctoral-level, biomedical professional career tracks (medicine, veterinary medicine, dentistry, optometry, or pharmacy).

Students were classified as having interest in biomedical fields of study at baseline (and thus included in the initial sample for this study) if they reported that they were pursuing a biomedical major on the baseline questionnaire, or if they checked any of the five pre-professional career boxes. Students who indicated interest in multiple majors were classified as having interest in the biomedical fields if any of their majors was biomedical in nature. Students who were undecided or had not declared a major at baseline were discussed on a case-by-case basis, using their interview responses to help classify them.

#### Determining pipeline retention through graduation

To classify whether students remained in the biomedical pipeline through graduation, we examined whether or not students pursued biomedical fields of study throughout college. To determine this, we examined students’ majors at graduation and classified them as biomedical or not using a scheme developed in previous research (*17*). If students had a biomedical major at graduation, they were classified as having remained in the biomedical field. Alternatively, if students had a nonbiomedical major at graduation, but had indicated interest in one of the five pre-professional career tracks at baseline, and were still pursuing one of these tracks at the time they were interviewed (e.g., a student who did a psychology major but retained plans throughout college to attend medical school), we classified them as remaining in biomedical fields of study. Students who did not clearly fall into one of these categories were classified on a case-by-case basis, using institutional data regarding their course-taking and their history of declared majors throughout college, as well as the information from their interviews.

We also confirmed whether students still studying biomedical fields had graduated college. Of the 1001 students whom we determined remained in the biomedical pipeline until graduation, 997 (99.6%) were classified as likely to have graduated college. We found clear evidence of graduation for 988 (98.7%) (966 had graduation records from the focal university, 10 had other evidence suggesting graduation from the focal university, and 12 had evidence of graduation from other universities). There were nine students for whom we could not find evidence of graduation, but our available data indicated that they were on track for graduation at the time of their interviews; we presumed that they had transferred to finish their degrees. There were four students whom we confirmed did not graduate college at the time of our analysis (two were still enrolled in college, two had dropped out); they were determined to have “leaked out” of the STEM pipeline. Of the 192 students who left biomedical fields of study throughout college (and thus had already leaked out of the pipeline), we found clear evidence of graduation for 188, and there were two students for whom we could not find evidence of graduation but we presumed that they had transferred and graduated based on available data. There were two students still enrolled in college.

#### Determining pipeline retention through career plans

To classify whether students continued to pursue biomedical career plans, we used the interviews with the 997 students who graduated with a biomedical degree and examined each student’s 10-year career goal. We then classified that career goal according to whether or not it was biomedical. We developed a classification scheme for this purpose using data from the O*Net Career Database (*23*). This database provides data about the most common career paths pursued in the United States, based on a taxonomy of over 1100 careers created by the U.S. Department of Labor and the Bureau of Labor Statistics (*24*). To create the database, the Department of Labor and National Center for O*Net Development worked with a consulting group to conduct a statistically random sample of businesses expected to employ workers in the careers of interest as well as a statistically random sample of workers who were employed by those businesses. This database includes much information about each career in the taxonomy; of interest in this study were the estimates of the amount of knowledge required for particular career paths in different categories, measured on a 0 to 100 scale. These estimates are based on ratings from incumbents in the professions.

A student’s career was classified as “biomedical” if the knowledge score in either the “biology” or “medicine” category for the corresponding career in the O*Net database was 60 or higher. Careers with scores between 45 and 60 in either category were discussed case-by-case by the authors and classified as biomedical or not (see Supplementary Text for career classification scheme). We added a general “biomedical career” category and a general “nonbiomedical career” category to our classification scheme to capture students who gave vague long-term career plans that could not be matched clearly to specific careers in the O*Net database. If students indicated two possible career paths, we classified the student as biomedical if at least one proposed career was biomedical.

As a second check of our classifications, if a student intended to pursue a career that was classified by O*Net as nonbiomedical, we then reviewed that student’s interview transcript to examine their future plans and examine whether they might still be pursuing substantive biomedical work as part of the career. If they were, we overruled the O*Net classification and classified the student as pursuing a biomedical career (e.g., if a student was going to obtain a Ph.D. in biomedical engineering and then become an entrepreneur to start a biotechnology company, we classified the student’s career as biomedical despite O*Net classifying most business-related occupations as nonbiomedical).

A small number of students did not provide clear career plans (e.g., they said they were undecided, or they wanted to do something broad such as “help people”). These students were discussed on a case-by-case basis using all available data (their course-taking and major plans throughout college, and their interview transcripts). We took an approach of assuming students were remaining in biomedical careers unless they indicated otherwise, given that they had expressed baseline interest in biomedical fields and pursuing biomedical coursework during college.

Of the 997 students who graduated with biomedical degrees, 921 retained biomedical career plans and constituted the “within-pipeline” sample for the remainder of the analysis (see supplementary tables for details of this sample).

#### Classifying educational requirements for careers

For the 921 students who remained in the pipeline and pursued biomedical careers, we classified their career educational aspirations at both baseline and the point at which they were interviewed. Aspirations could be classified into one of three categories at each time point based on the amount of future education students planned to pursue: bachelors level, masters level, or doctoral level and higher (higher would be a dual substantive degree such as an M.D. and a Ph.D., or an M.D. and an M.P.H. degree). Students could also be classified as “undecided” if they gave no indications of their level of career aspirations at baseline (no students were fully undecided by the time of graduation, as they all reported at least vague future plans in their interviews).

To determine the amount of future education associated with students’ plans, we used education and training data from the O*Net database. For each career in the database, there is O*Net data regarding the proportions of job incumbents for a particular profession who reported that a certain type of education is required for that career (*24*). Employees could choose from 12 educational categories, ranging from less than a high school degree to post-doctoral training; we binned these into three categories corresponding to the most frequent educational aspirations students reported in our sample: bachelors level and below, masters level or combined bachelors-professional degrees, and doctoral level or above. Some O*Net categories were too broad to make clear classifications (e.g., environmental scientists and specialists) and for those we chose to determine educational aspirations on a case-by-case basis rather than use the O*Net data as a deciding factor. We also did this for the two “general” career categories that we created (see Supplementary Text for complete list of classifications and list of careers for which we made case-by-case decisions).

Using our classification scheme, we determined which of the three categories was most frequently reported for a given career and classified the career’s typical educational aspirations accordingly. Then, we made preliminary classifications of students’ baseline and final career aspirations using this classification scheme. However, to ensure that our categorizations were accurate, we also considered each student’s interview data before making a final classification of a given student’s aspirations. That is, we examined students’ interview transcripts to determine if they had stated specific plans for graduate school pursuits either at baseline or after graduating. If students reported graduate school plans that contradicted the results from the O*Net database, we used students’ responses instead of what O*Net suggested as a designated level of education (e.g., if a student’s career was classified as masters-level but they stated that they planned to get a Ph.D., we classified them as having doctoral-level aspirations).

Similar to career titles, in some cases, students did not provide specific future career educational aspirations at baseline or at the follow-up. In these cases, we made decisions about students’ baseline and final aspirations on a case-by-case basis using their interview responses. We also used a rule of assuming that students’ educational aspirations were bachelors-level in the absence of additional information, because all of the students were enrolled in college (or had graduated) at the point of being interviewed. Supplementary Text reports the breakdown of students’ baseline and final career aspirations, overall, as a function of gender and ethnicity separately, and as a function of the intersection of gender and ethnicity.

#### Determining whether or not career plans changed

For all students who remained in the biomedical pipeline through to the pursuit of biomedical careers, we classified whether or not their career plans had changed based on their answer to the question “Are your career plans different now compared to what they were when you started introductory biology?” Supplementary Text reports the numbers and gender/ethnic breakdown of students whose plans changed (*N* = 422) and did not change (*N* = 499).

#### Coding type of career plan change

For the 422 students who indicated that their career plans had changed, we classified the type of change into one of three categories based on their educational aspirations: changed to a career requiring the same amount of education, changed to a career requiring fewer years of education, or changed to a career requiring more years of education. We did this by comparing students’ career aspirations at baseline to their career aspirations as stated in their interviews.

There were two exceptions to this rule. First, some students had initially been undecided at baseline about their aspirations, and in their interviews, they stated that they developed a clearer sense of what they wanted to do over the course of college. These students were classified as experiencing a same-level change in aspirations because they did not raise or lower their expected amount of education. Second, a small number of students (*N* = 13) who changed career plans indicated that they wanted to pursue doctoral degrees plus substantive additional degrees, whereas at baseline they had wanted to pursue only doctoral-level degrees. We classified these students as having gone up in their educational aspirations, because they had raised their intended years of future education from their initial pursuits.

Table 1 reports the breakdown of this coding overall and provides examples of students’ interview responses corresponding to each type of change; these quotations have been edited in minor ways for grammar and readability. Supplementary tables report how these categories break down by gender and ethnicity, the breakdown of students’ baseline and final career aspirations as a function of whether or not their career plans changed (overall and by gender and ethnicity), and a specific case study analysis of the final careers chosen by students who initially intended to pursue medical school but then lowered their aspirations for future education (overall and by gender).

### Classifying career plan changes as being due to disenchantment or attraction

For the 422 students who changed their career plans, we classified whether they interpreted this change in terms of disenchantment versus attraction. Students were classified as interpreting the change due to disenchantment if they indicated that their change in plans was primarily due to negative perceptions of their original field of study or career plan that caused them to leave; they were classified as interpreting the change due to attraction if they indicated that positive perceptions of their new field of study or career plan attracted them toward it. Students could be classified into a “both” category if they described both disenchantment and attraction as influencing their decision to change plans. One of three coders classified each student’s response into one of the categories or designated the response as too vague to classify (𝜅s = 0.72 to 0.83 between pairs of coders, based on cross-coding 13 to 20% of responses); disagreements were resolved by the first author.

The critical distinction in classifying disenchantment versus attraction was whether students discussed their change in plans as being influenced by something about their original major/career plan or their new major/career plan. The reasons behind students experiencing disenchantment or attraction could be external or internal to the student. That is, a student might report disenchantment because she felt that she was not capable of meeting admissions requirements for medical school, or because a teacher encouraged her to drop a class after she failed a test.

The goals of this paper were to consider how many students reflected on their change in plans within STEM fields in terms of being caused by potential challenges they experienced; hence, we were interested in examining any students who reported disenchantment, even if those students also reported some attraction as motivating their change in plans. We therefore chose to classify students in the “both disenchantment and attraction” group along with students in the “disenchantment only” group for the primary analyses. However, the significant effects in the within-pipeline analyses did not change if we classified the “both” students in with the “attraction only” group, analyzed them as a separate group, or removed them from analyses.

Table 1 reports the overall classification of disenchantment versus attraction in the sample and provides examples of students’ interview responses corresponding to each explanation; these quotations have been edited in minor ways for grammar and readability. In Supplementary Text, there are tables breaking down students’ explanations by type of change and by gender and ethnicity.

#### Demographic data

Demographic information regarding gender and URM status was collected using a combination of self-report and institutional records.

### Analytic strategy

We examined frequencies of students’ responses to the variables we coded from their interview and institutional data, and we conducted chi-square tests of independence to determine whether there was overall heterogeneity in the co-occurrence of different categories of change across demographic categories. In the case of significant chi-square tests using the type of career plan change variable, which had more than one degree of freedom, we conducted follow-up one-degree-of-freedom chi-square tests examining each specific category within the broader table (i.e., each specific type of career plan change) as a function of the other predictor variable (women versus men, URM students versus majority students, or disenchantment versus attraction).

Full output for all analyses can be found in supplementary tables.

## SUPPLEMENTARY MATERIALS

Supplementary material for this article is available at http://advances.sciencemag.org/cgi/content/full/7/18/eabe0985/DC1

## References

[1] A. L. Griffith, Persistence of women and minorities in STEM field majors: Is it the school that matters? Econ. Educ. Rev. 29, 911–922 (2010).

[2] T. R. Sass, *Understanding the STEM Pipeline* (American Institutes for Research, 2015).

[3] A. V. Maltese, R. H. Tai, Pipeline persistence: Examining the association of educational experiences with earned degrees in STEM among U.S. students. Sci. Educ. 95, 877–907 (2011).

[4] U.S. Department of Education, *A Leak in the STEM Pipeline: Taking Algebra Early* (U.S. Department of Education, 2018).

[5] J. C. Blickenstaff, Women and science careers: Leaky pipeline or gender filter? Gend. Educ. 17, 369–386 (2005).

[6] M. A. Cannady, E. Greenwald, K. N. Harris, Problematizing the STEM pipeline metaphor: Is the STEM pipeline metaphor serving our students and the STEM workforce? Sci. Educ. 98, 443–460 (2014).

[7] H. Metcalf, Stuck in the pipeline: A critical review of STEM workforce literature. InterActions UCLA J. Educ. Inf. Stud. 6, (2010).

[8] P. Boyle, U.S. physician shortage growing (2020); www.aamc.org/news-insights/us-physician-shortage-growing.

[9] J. Eccles, Expectancies, values, and academic behaviors, in *Achievement and Achievement Motives: Psychological and Sociological Approaches*, J. T. Spence, Ed. (Freeman, 1983), pp. 75–146.

[10] J. S. Eccles, A. Wigfield, From expectancy-value theory to situated expectancy-value theory: A developmental, social cognitive, and sociocultural perspective on motivation. Contemp. Educ. Psychol. 61, 101859 (2020).

[11] E. Seymour, N. M. Hewitt, *Talking About Leaving* (Westview Press, 1997).

[12] E. Seymour, A. B. Hunter, *Talking About Leaving Revisited* (Springer, 2019).

[13] E. Q. Rosenzweig, J. M. Harackiewicz, C. A. Hecht, S. J. Priniski, E. A. Canning, Y. Tibbetts, M. W. Asher, J. S. Hyde, College students’ reasons for leaving biomedical fields: Disenchantment with biomedicine or attraction to other fields? J. Educ. Psychol. 13, 351–369 (2020). 10.1037/edu0000456 PMC7989803 33776138

[14] National Science Board, *Science and Engineering Indicators* (2018); www.nsf.gov/statistics/2018/nsb20181/.

[15] M. J. Chang, J. Sharkness, S. Hurtado, C. B. Newman, What matters in college for retaining aspiring scientists and engineers from underrepresented racial groups. J. Res. Sci. Teach. 51, 555–580 (2014).

[16] S. Cheryan, S. A. Ziegler, A. K. Montoya, L. Jiang, Why are some STEM fields more gender balanced than others? Psychol. Bull. 143, 1–35 (2017). 27732018 10.1037/bul0000052

[17] E. A. Canning, J. M. Harackiewicz, S. J. Priniski, C. A. Hecht, Y. Tibbetts, J. S. Hyde, Improving performance and retention in introductory biology with a utility-value intervention. J. Educ. Psychol. 110, 834–849 (2020). 10.1037/edu0000244 PMC6168083 30294006

[18] J. Eccles, Who am I and what am I going to do with my life? Personal and collective identities as motivators of action. Educ. Psychol. 44, 78–89 (2009).

[19] A. B. Diekman, M. Steinberg, E. R. Brown, A. L. Belanger, E. K. Clark, A goal congruity model of role entry, engagement, and exit: Understanding communal goal processes in STEM gender gaps. Person. Soc. Psychol. Rev. 21, 142–175 (2017). 10.1177/1088868316642141 27052431

[20] A. J. Starmer, M. P. Frintner, K. Matos, C. Somberg, G. Freed, B. J. Byrne, Gender discrepancies related to pediatrician work-life balance and household responsibilities. Pediatrics 144, e20182926 (2019). 31506304 10.1542/peds.2018-2926

[21] J. M. Harackiewicz, E. A. Canning, Y. Tibbetts, C. J. Giffen, S. S. Blair, D. I. Rouse, J. S. Hyde, Closing the social class achievement gap for first-generation students in undergraduate biology. J. Educ. Psychol. 106, 375–389 (2014). 25049437 10.1037/a0034679 PMC4103196

[22] J. M. Harackiewicz, E. A. Canning, Y. Tibbetts, S. J. Priniski, J. S. Hyde, Closing achievement gaps with a utility-value intervention: Disentangling race and social class. J. Pers. Soc. Psychol. 111, 745–765 (2016). 26524001 10.1037/pspp0000075 PMC4853302

[23] National Center for O*NET Development, *O*NET OnLine* (2020); www.onetonline.org.

[24] National Center for O*Net Development, *O*Net Data Collection Overview* (2020); www.onetcenter.org/dataCollection.html.

